# Mitochondrial Dysfunction in Human Leukemic Stem/Progenitor Cells upon Loss of RAC2

**DOI:** 10.1371/journal.pone.0128585

**Published:** 2015-05-27

**Authors:** Marta E. Capala, Henny Maat, Francesco Bonardi, Vincent van den Boom, Jeroen Kuipers, Edo Vellenga, Ben N. G. Giepmans, Jan Jacob Schuringa

**Affiliations:** 1 Department of Experimental Hematology, Cancer Research Center Groningen, Groningen, the Netherlands; 2 Department of Cell Biology, University Medical Center Groningen, University of Groningen, Groningen, the Netherlands; French Blood Institute, FRANCE

## Abstract

Leukemic stem cells (LSCs) reside within bone marrow niches that maintain their relatively quiescent state and convey resistance to conventional treatment. Many of the microenvironmental signals converge on RAC GTPases. Although it has become clear that RAC proteins fulfill important roles in the hematopoietic compartment, little has been revealed about the downstream effectors and molecular mechanisms. We observed that in BCR-ABL-transduced human hematopoietic stem/progenitor cells (HSPCs) depletion of RAC2 but not RAC1 induced a marked and immediate decrease in proliferation, progenitor frequency, cobblestone formation and replating capacity, indicative for reduced self-renewal. Cell cycle analyses showed reduced cell cycle activity in RAC2-depleted BCR-ABL leukemic cobblestones coinciding with an increased apoptosis. Moreover, a decrease in mitochondrial membrane potential was observed upon RAC2 downregulation, paralleled by severe mitochondrial ultrastructural malformations as determined by automated electron microscopy. Proteome analysis revealed that RAC2 specifically interacted with a set of mitochondrial proteins including mitochondrial transport proteins SAM50 and Metaxin 1, and interactions were confirmed in independent co-immunoprecipitation studies. Downregulation of SAM50 also impaired the proliferation and replating capacity of BCR-ABL-expressing cells, again associated with a decreased mitochondrial membrane potential. Taken together, these data suggest an important role for RAC2 in maintaining mitochondrial integrity.

## Introduction

Hematopoiesis is a hierarchical process, initiated by hematopoietic stem cells (HSCs) that reside within specialized regions of the bone marrow, termed the niche [1,2]. A constant crosstalk between an HSC and its microenvironment provides signals that maintain the HSC in a quiescent state and regulate its proliferation and differentiation, crucial both for the homeostasis of the hematopoietic system and stress hematopoiesis [3–9]. The hierarchical organization of the healthy hematopoietic system is to a certain extent maintained upon malignant transformation. Mouse xenograft models have shown that leukemic cells have a phenotypic hierarchy, and that only a subpopulation of malignant cells is able to recapitulate the disease in recipient animals [10,11]. This ability to initiate, maintain and serially propagate leukemia in vivo is the hallmark property of leukemic stem cells (LSCs) [10]. Similarly to their healthy counterparts, LSCs are also found within specialized bone marrow niches, and they utilize this microenvironment to maintain a relatively quiescent state. Consequently, LSCs are able to escape the cytotoxic effects of chemotherapy and give rise to the relapse of the disease, which occurs in a large majority of acute myeloid leukemia (AML) patients [12,13]. In chronic myeloid leukemia (CML), the dormant LSCs are largely independent on the BCR-ABL signaling and therefore cannot be eradicated by BCR-ABL tyrosine kinase inhibitors (TKIs), so that disease often reoccurs upon discontinuation of TKI treatment [14–17]. It is postulated that the disruption of the LSC-niche interactions leading to the egress of LSCs from their microenvironment would facilitate targeting of those cells [18,19]. Therefore, identification of the key components of the LSC niche will be instrumental for the ultimate eradication of leukemia.

Proteins of the RAC family have been identified as crucial mediators of the interactions between hematopoietic stem cells (HSCs) and their microenvironment [20,21]. These small GTPases act as molecular switches, cycling between an inactive GDP-bound state and an active state in which they are GTP-bound. RACs are activated by various signaling events from the cell surface, such as activation of tyrosine kinase receptors, G protein-coupled receptors, and cell-to-cell contacts. These in turn activate several downstream targets, including cytoskeleton rearrangements [22–25]. Consequently, RAC proteins have a critical role for the biology of HSCs, as they have been implicated in such processes as migration, homing and retention of HSCs in the bone marrow [20,26,27]. The RAC family consists of RAC1, RAC2 and RAC3, which show tissue-specific expression distribution. RAC1 and RAC2 are both expressed in the hematopoietic system and despite high sequence homology (90%) both unique and overlapping functions of these two proteins have been proposed in murine hematopoiesis [20,26,28]. Next to its crucial role in normal hematopoiesis, RAC activity has been implicated in the disease initiation and maintenance in various murine leukemia models, including BCR-ABL and MLL-AF9-driven transformation [29–32]. In accordance with that, inhibition of RAC activity has been proven a successful strategy to target primary human AML and CML cells *in vitro* and *in vivo* [29,32,33]. Despite the growing understanding of their role in murine models, our insight into molecular mechanisms by which RAC proteins contribute to the development of human leukemia remains limited.

Here, using RNA-interference we observed that BCR-ABL-transduced human hematopoietic stem/progenitor cells (HSPCs) critically depended on RAC2 for their long-term growth. Assessment of the mitochondrial membrane potential in RAC2-depleted cells indicated mitochondrial dysfunction, which coincided with ultrastructural abnormalities identified in RAC2-deficient BCR-ABL cells by electron microscopy. Moreover, we performed a proteomics screen to identify RAC1- and RAC2-specific interaction partners and found several mitochondrial proteins to be specifically associated with RAC2, including SAM50 and Metaxin1. We propose that RAC2 is necessary for the proper mitochondrial function in BCR-ABL-expressing HSPCs and that inhibiting RAC2 could aid in the targeting of those primitive leukemic cells.

## Materials and Methods

### Primary cell isolation and culture conditions

Neonatal cord blood (CB) was obtained from healthy full-term pregnancies after informed consent in accordance with the Declaration of Helsinki from the obstetrics departments of the University Medical Centre Groningen (UMCG) and Martini Hospital Groningen, Groningen, The Netherlands. Donors were informed about procedures and studies were performed with CB by an information sheet that was read and signed by the donor, in line with regulations of the Medical Ethical Committee of the UMCG. All protocols were approved by the Medical Ethical Committee of the UMCG. After separation of mononuclear cells with lymphocyte separation medium (PAA Laboratories), CD34^+^ cells were isolated using a magnetically activated cell sorting (MACS) CD34 progenitor kit (Miltenyi Biotech). For the MS5 co-culture experiments, cells were grown in Gartner’s medium consisting of α-modified essential medium (α–MEM; Fisher Scientific Europe) supplemented with 12.5% heat-inactivated fetal calf serum (Lonza), 12.5% heat-inactivated horse serum (Invitrogen), 1% penicillin and streptomycin, 2mM glutamine (all from PAA Laboratories), 57.2 μM β-mercaptoethanol (Merck Sharp & Dohme BV) and 1μM hydrocortisone (Sigma-Aldrich Chemie B.V.). Blast crisis chronic myeloid leukemia (BC CML) blasts from peripheral blood cells from an untreated patient with BC CML (patient 2007–048, with the t(9;22) translocation) were obtained and studied after informed consent in accordance with the Declaration of Helsinki, and the protocol was approved by the Medical Ethical Committee of the UMCG. Donors were informed about procedures and studies performed with AML cells by an information sheet that was read and signed by the donor, in line with regulations of the Medical Ethical Committee of the UMCG. BC CML mononuclear cells were isolated by density gradient centrifugation and CD34^+^ cells were stained using CD34-PE antibody (BD Biosciences) and selected by sorting on a MoFLo (DakoCytomation). BC CML co-cultures were expanded in Gartner’s medium supplemented with 20ng/mL interleukin 3 (IL-3; Gist-Brocades), granulocyte-colony stimulating factor (G-CSF; Rhone-Poulenc Rorer) and thrombopoietin (TPO; Kirin).

### Cell lines and culture conditions

293T embryonic kidney cells (ACC-635 DSMZ) and PG13 packaging cells (ATCC CRL-10686) were grown in DMEM medium with 200mM glutamine (BioWhittaker) supplemented with 10% FSC and 1% penicillin and streptomycin. K562 myelogenous leukemia cells (ACC-10, DSMZ) and TF-1 erythroleukemic cells (ACC-334, DSMZ) were grown in RPMI medium with 200mM glutamine (BioWhittaker) supplemented with 10% FCS, and 1% penicillin and streptomycin, and for TF-1 cells with 5 ng/ml granulocyte-macrophage colony stimulating factor (GM-CSF; Genetics Institute, Cambridge, MA, USA). MS-5 murine stromal cells (ACC-441, DSMZ) were grown in αMEM with 200mM glutamine (BioWhittaker) supplemented with 10% FCS and 1% penicillin and streptomycin.

### Retro- and lentivirus generation and transduction

Stable PG13 producer cell lines of BCR-ABL retroviral constructs were generated and used as published previously [34]. Supernatants from the PG13 cells were harvested after 8 to 12 hours of incubation in human progenitor growth medium (HPGM; Cambrex) before the retroviral transduction rounds and passed through 0.45 μM filters (Sigma-Aldrich). Before the first transduction round, CD34^+^ CB cells were pre-stimulated for 48 hours in HPGM supplemented with 100ng/ml stem cell factor (SCF), FLT3 Ligand (Flt3L; both from Amgen) and TPO. Three rounds of transduction were performed on retronectin-coated 24-well plates in the presence of the same cytokines as for pre-stimulation and 4 μg/mL polybrene (Sigma-Aldrich). During the last round of retroviral transduction with BCR-ABL, lentiviral shRNA particles were also added as described below.

Short hairpin RNA (shRNA) sequences targeting RAC1 (AACCTTTGTACGCTTTGCTCA), RAC2 (AAGGAGATTGACTCGGTGAAA), or SAM50 (GCGGAATGTTGGTACCCATTG) were ligated into pHR’trip vector using AcsI and SbfI restriction sites. For the control, scrambled (Scr) shRNA sequence was used. 293T embryonic kidney cells were transfected using FuGENE6 (Roche) with 3 μg pCMV Δ8.91, 0.7 μg VSV-G, and 3 μg of pHR’trip vector constructs (shSCR, shRAC1, shRAC2 or shSAM50). After 24 hours medium was changed to HPGM and after 12 hours supernatant containing lentiviral particles was harvested and either stored at -80°C or used fresh for transduction of target cells. BCR-ABL-transduced CD34^+^ CB cells were subjected to 1 round of transduction with lentiviral particles, together with the last round of retroviral transduction, in the presence of prestimulation cytokines and 4 μg/mL polybrene (Sigma) on retronectin-coated 24-well plates. After transduction, transduced green fluorescent protein (GFP)-positive (indicative of transduction with shRNA-containing vectors), truncated nerve growth factor receptor (NGFR)-positive (indicative of transduction with BCR-ABL-containing vector) or double-positive cells were sorted on a MoFlo sorter (Dako Cytomation). BC CML CD34^+^ cells were transduced as described previously [35]. Briefly, transductions were performed in 3 consecutive rounds of 8 to 12 hours with lentiviral supernatant supplemented with 10% FCS, IL-3, granulocyte-colony stimulating factor (G-CSF; Rhone-Poulenc Rorer, Amstelveen, The Netherlands) and TPO (20ng/m each) and polybrene (4 μg/ml) on a retronectin-coated 24-wells plate. After transduction, transduced green fluorescent protein (GFP)-positive (indicative of transduction with shRNA-containing vectors) were sorted and used to initiate co-cultures.

For transient retroviral transduction, RAC1 and RAC2 were cloned into MiGR1-Avi-IRES2-EGFP-BirA vector using AgeI and MluI restriction sites. Plasmid RNA was purified using RNeasy kit (Qiagen) according to manufacturer’s protocol. Retroviral particles were produced by transfection of embryonic kidney 293T cells. Briefly, 293T cells were cultured in DMEM (Lonza) supplemented with 10% FCS and 1% P/S in gelatine-coated flasks. The following day, medium was refreshed and 2–6 hr thereafter cells were (co)transfected with packaging plasmid pCLampho (3.5 μg) and vector constructs for BirA, Avi-RAC1 orAvi-RAC2 (3.5 μg) in the presence of FuGENE HD (Promega). After 24 hr medium was changed to RPMI. The next day retroviral supernatant was collected, passed through 0.45 um filter (Millipore, Amsterdam, The Netherlands) and directly used for stable transduction. K562 cells (0.5x10^6^) were transduced with retroviral supernatant of either Avi-RAC1 or Avi-RAC2 or BirA, which served as a control, in two consecutive rounds together with 4 ug/mL of polybrene (Sigma-Aldrich). After transduction, GFP positive cells were sorted by Fluorescence-Activated Cell Sorting (FACS) (MoFlo-Astrios, BeckmanCoulter).

### Long-term cultures on stroma, CFC and LTC-IC assay

After sorting, 5x10^3^ BCR-ABL cells were plated onto T25 flask pre-coated with MS5 stromal cells in 5 mL of Gartner’s medium in duplicate. Co-cultures were kept at 37°C and 5% CO_2_ and cells were demi-depopulated weekly for analysis. For the inhibition of Rac activity, NSC2766 (NSC; Calbiochem, VWR, Amsterdam, The Netherlands) was added to the co-culture medium to the final concentration of 20μM, 40μM or 100μM. For the microscopical evaluation of cobblestone cells, 2x10^3^ BCR-ABL cells shSCR (short hairpin scrambled oligonucleotides)- or shRAC2-transduced were plated into T12.5 flasks pre-coated with stromal MS5 cells in 3 mL Gartner’s medium. Co-cultures were evaluated daily and images of cobblestones were acquired on Leica DMIL inverted phase microscope. Images were then opened in CorelDRAW X5 software (Corel Software), overlain with a grid and cells forming an individual cobblestone were counted. CFC assays were performed as previously described [36]. For the LTC-IC assay, BCR-ABL cells were plated in the range of 2 to 486 cells per well in a 96-well plate using Gartner’s medium. Methylcellulose (StemCell Technologies) supplemented with 20 ng/mL of IL-3, 20 ng/mL of interleukin-6 (IL-6; Gist-Brocades), 20 ng/mL of G-CSF, 20ng/mL of c-kit ligand (Amgen), and 6 U/mL of erythropoietin (Epo; Cilag) was added at week 5. Two weeks later, wells containing CFCs were scored as positive and the LTC-IC frequency was calculated using L-Calc software (StemCell Technologies).

### Mitochondrial mass and membrane potential

Mitochondrial mass was determined by staining the cells with MitoTracker Red FM dye (Life Technologies) according to the manufacturer’s recommendations. 5x10^5^ cells were incubated with 20 nM MitoTracker for 30 min at 37°C, washed in PBS, and analyzed by FACS. Mitochondrial membrane potential was measured by flow cytometry using hexamethylindodicarbocyanine iodide (DilC1) (Molecular Probes) as described previously [37]. Briefly, 5x10^5^ cells were incubated with 50 ng/ml DilC1 for 30 min at 37°C, washed twice in PBS, and DilC1 fluorescence was analyzed by FACS.

### Electron microscopy

BCR-ABL and shSCR- or shRAC2-double transduced HSPCs were collected after 7 days of co-culture on stroma, pelleted and subsequently fixed in 2% glutaraldehyde in 0.1 M sodiumcacodylate buffer for 24 h at 4°C. After postfixation in 1% osmiumtetroxide/1.5% potasiumferrocyanide (2 hr at 4°C), cells were dehydrated using ethanol and embedded in EPON using standard procedures [38]. 60nm sections were cut and contrasted using 2% uranylacetate in methanol followed by Reynolds leadcitrate. Images were taken with a Zeiss Supra55 in STEM mode at 29 KV using an external scan generator (Fibics) yielding large area scans containing many cells at 3nm/pixel resolution. Examples of acquired images can be found online (for control cells: http://figshare.com/s/2de8ed72e8e711e492b606ec4bbcf141; for RAC2-depleted cells: http://figshare.com/s/9ec695f2e8e311e492bb06ec4b8d1f61). 131 control cells and 139 shRAC2-transduced cells were analyzed for the presence of mitochondrial abnormalities.

### Pull-down assays and immunoblotting

Sorted K562 cells transduced with BirA, Avi-RAC1 or Avi-RAC2 constructs were pelleted (each 10^7^ cells) and washed once with cold PBS, in line with our previously published studies [39]. Cell lysis was performed using an ice-cold buffer consisting of 20 mM Hepes pH 7.5 (Life Technologies), 0.1% Tween 20 (Merck), 0.5% Triton X-100, 150 mM NaCl, 0.1% Sodium deoxycholate (all from Sigma-Aldrich) and (freshly added) protease inhibitors (1 mM DTT, 250x CLAP, 0.1 mM PMSF, all from Sigma-Aldrich) while incubated for 30 min on a rotating wheel at 4°C. Cell fragments were removed by centrifugation for 15 min at 14.000 rpm at 4°C. Magnetic Dynabeads M-280 Streptavidin (Invitrogen) were equilibrated in lysis buffer and 1mg beads were added to each total cell lysate and incubated overnight at 4°C on a rotating wheel. Alternatively, immunoprecipitation with anti-SAM50 antibody and Dynabeads Protein A (invitrogen) was performed according to manufacturer’s instructions. Beads were separated by the use of a magnetic rack and washed six times in the lysis buffer. In an alternative approach, sorted K562 cells transduced with GFP-RAC1 or GFP-RAC2 constructs were pelleted (each 10x10^6^ cells) and washed once with cold PBS. Empty vector-GFP-transduced cells were used as a control. Cell lysate preparation was performed as described above and pull-down assay was performed using GFP-Trap beads from ChromoTek according to manufacturer’s instructions. For both streptavidin-based and GFP-based pull-down assays, bound fractions (B) were eluted from the beads by boiling for 5 min in Leammli sample buffer and ½ of total cell lysate (T), bound (B) and non-bound (NB) fractions were analyzed by Western blot.

Western blot analysis was performed according to standard protocols. Antibody against RAC1 (clone 23A8) and RAC2 were obtained from Millipore, anti-GFP from Abcam and Strepatividin-HRP conjugate antibody from PerkinElmer. Antibodies against SAM50 and Metaxin 1 were kindly provided by Dr. V. Kozjak-Pavlovic (University of Wϋrzburg, Wϋrzburg, Germany). Secondary antibodies (rabbit-anti-mouse-HRP and goat-anti-rabbit-HRP) were purchased from Dako Cytomation and used in 1:3000 dilutions. Binding of antibodies was detected by chemiluminescence, according to the manufacturer’s instructions (Roche Diagnostics).

### Mass spectrometry analysis

Bound fraction samples for BirA, Avi-RAC1 and Avi-RAC2 were separated on a 4–12% NuPAGE pre-cast gel (Invitrogen), stained with Coomassie blue G250 and subsequently destained overnight. Gel bands were excised into 9/10 gel slices for in-gel trypsin digestion. Each slice was further cut into 1 mm pieces and completely destained using 50 mM ammonium bicarbonate (ABC) in 50% acetonitrile (ACN). A reduction and alkylation step was performed to block cysteins. Briefly, 10 mM DTT in 50mM ABC was added to the gel pieces and incubated for 45 min at 55°C. Next, 55 mM iodoacetamide in 50 mM ABC was added and incubated for 30 min at room temperature. Gel pieces were dehydrated and allowed to dry, after which they were re-swelled in 10 ng/ul trypsin solution in 40 mM ABC and 10% ACN at 37°C overnight. Peptides were fully extracted by adding 2% trifluoroacetic acid (TFA) to the gel pieces, proceeded by extraction with 33% ACN, 1.3% TFA and followed by 66% ACN, 0.7% TFA. Extracted peptides were combined and then completely dried in a SpeedVac centrifuge. Peptide mixtures were further processed for mass spectrometry (LC-MS/MS) analysis on a linear ion trap LTQ-Orbitrap XL (Thermo Scientifics). Two search engines were used: Mascot (version 2.1.04) and X! Tandem (CYCLONE; 2010.12.01.1) Peptide identifications were accepted with a probability above 90% and proteins were accepted when the probability score was higher than 90% and at least one unique peptide was identified (Scaffold software; version 3.4.9). Identified proteins were analyzed by DAVID functional annotation tool.

### Fluorescent microscopy

N-terminal GFP fusion constructs of RAC1 and RAC2 were cloned into pRRL construct and lentivial particle generation was performed as described in”Retro- and lentivirus generation and transduction” section. TF-1 cells were subjected to 1 round of transduction with lentiviral particles in the presence of 4 μg/mL polybrene (Sigma) on retronectin-coated 12-well plates and GFP^+^ cells were sorted on a MoFlo sorter. Cells were then cytospun and fixated and images were acquired with a Leica SP2 AOBS confocal microscope, 63x objective. Images were analyzed with ImageJ software.

### RNA extraction and Real-time PCR analysis

RAC1 or RAC2 expression was assessed by quantitative real-time PCR (qRT-PCR). Total RNA was isolated using RNeasy kit (Qiagen) following the manufacturer’s recommendations. After reverse transcription using M-MuLV reverse transcriptase (Fermentas), according to manufacturer’s instructions, aliquots of cDNA were real-time amplified using iQ SYBR Green mix (Bio-Rad) on a MyIQ thermocycler (BioRad). RAC1 and RAC2 primer sets were obtained from Invitrogen. Expression was quantified using MyIQ software (Bio-Rad) and RPL27 expression was used to calculate relative expression levels of investigated genes according to standard curve method.

### Statistical analysis

All values are expressed as means ± standard deviation (SD). Student’s *t* test was used for all comparisons. Differences were considered statistically significant at *p*<0.05.

## Results

### Human BCR-ABL transduced cells critically depend on RAC2 signaling

**Fig 1:**
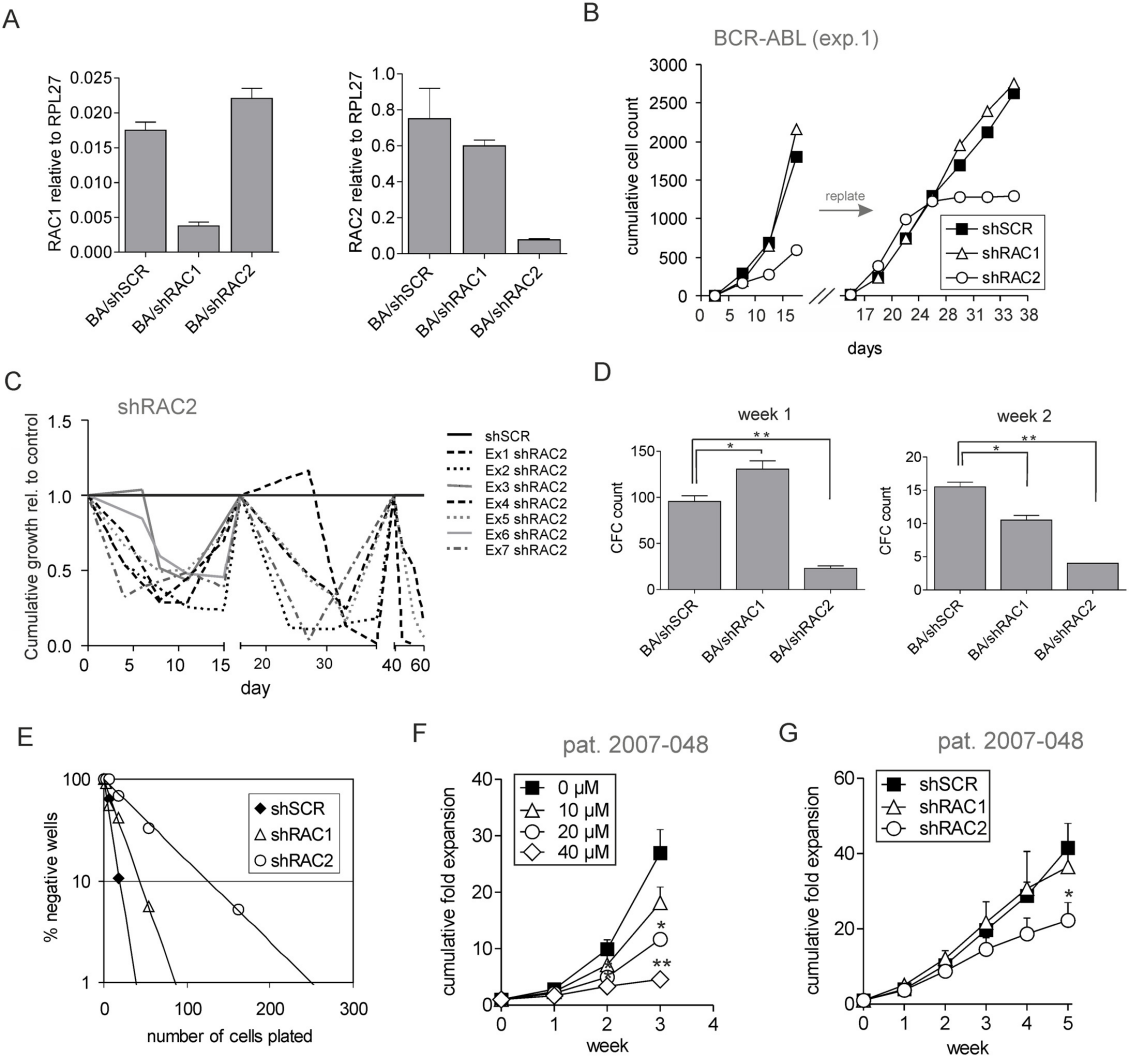
Human BCR-ABL-transduced HSPCs specifically depend on RAC2, but not RAC1 expression. (A) Cord blood (CB) CD34^+^ stem/progenitor cells were co-transduced with a retroviral construct for BCR-ABL and either a control scrambled shRNA vector (shSCR) or with RAC1/RAC2-targeting shRNA vectors (shRAC1 or shRAC2). Transduced cells were sorted, cultured on stroma for 10 days and used for RNA extraction. Quantitative PCR was performed to measure RAC1 (left panel) or RAC2 mRNA levels (right panel) normalized against RPL27 mRNA. (B) CB CD34^+^ stem/progenitor cells were co-transduced with BCR-ABL and either control shSCR or with shRAC1 or shRAC2. 5x10^3^ double-transduced cells were sorted per group and plated on MS5 stromal cells. Cultures were demi-depopulated on indicated days for analysis and replated as indicated. The cumulative cell growth is shown for a representative experiment out of 7 independent experiments. (C) Fold reduction of cumulative cell growth of BCR-ABL and shRAC2-double-transduced CD34^+^ CB cells in long-term co-culture as described in panel B, normalized to control cells. Cumulative expansion curves of 7 independent experiments are shown. (D) Suspension cells from MS5 co-cultures as described in panel B were analyzed for progenitor frequency by CFC assay at week 1 and week 2 of the co-culture. 10^4^ cells from each co-culture were plated in a CFC assay in methylcellulose in duplicate, and colonies were evaluated 2 weeks after plating. Total CFC numbers are shown from a representative of 5 independent experiments. Error bars indicate standard deviation. (E) LTC-IC frequencies were determined in limiting dilution on MS5 stromal cells. After 5 weeks of culture methylcellulose was added and colonies were scored two weeks later. Poisson statistics were used to calculate LTC-IC frequencies (p = 0.1 for shSCR vs shRAC1 and p = 0.0001 for shSCR vs shRAC2). (F) BC CML CD34^+^ cells (patient 2007–043) were sorted and plated on MS5 stroma. Cells were allowed to proliferate for 5 days after which RAC inhibitor was added to the following concentrations: 10μM, 20μM or 40μM. Co-cultures were demi-depopulated on indicated days for analysis. Cumulative cell count is shown as an average of 3 independent experiments. Error bars represent standard deviation. (G) BC CML CD34^+^ cells (patient 2007–043) were transduced with either control shSCR or with shRAC1 or shRAC2. 5x10^4^ transduced cells were sorted per group and plated on MS5 stromal cells; cultures were demi-depopulated weekly for analysis. Cumulative cell growth is shown as an average of 3 independent experiments. Error bars represent standard deviation. * *P*<0.05, ** *P*<0.01.

RAC GTPases are necessary for development and maintenance of leukemia in various murine models, including BCR-ABL-mediated transformation [30–32]. In order to specifically address the role of either RAC1 or RAC2 in human leukemia, we used shRNA interference strategy. We designed shRNA sequences that specifically and efficiently targeted mRNA of the designated RAC protein, with no effect on the expression of others (Fig 1A and S1 Fig). To investigate the importance of RAC signaling in human leukemic cells, we retrovirally transduced CD34^+^ CB cells to overexpress the BCR-ABL oncogene. Simultaneously, lentiviral transduction with shRNA-containing constructs was performed and double-transduced cells were sorted. Strikingly, RAC2-depleted cells reproducibly showed profound growth impairment within the first weeks of co-culture, which became even more apparent upon replating (Fig 1B and 1C). Moreover, progenitor frequencies in RAC2-depleted co-cultures were markedly reduced (Fig 1D) as well as of more primitive LTC-IC cells (1:57 vs. 1:11, p = 0.0001; Fig 1E). Notably, none of these phenotypes was observed in RAC1-depleted cells in which proliferation was comparable to controls, progenitor frequencies were initially higher and only mildly decreased at later time points, and LTC-IC frequencies were not significantly different than those of control cells (Fig 1B, 1D and 1E). Next, we investigated the importance of RAC signaling in primary human leukemic cells. To this end, we isolated CD34^+^ cells from the peripheral blood of a patient diagnosed with blast crisis CML (BC CML) and plated them in stromal co-cultures. Plated cells were allowed to expand and form cobblestones, after which the RAC inhibitor NSC23766 (NSC) was added to the co-cultures in concentrations of 10μM to 40μM. Treatment with RAC inhibitor resulted in a reduced expansion of BC CML cells in a dose-dependent manner. Co-cultures treated with 20μM and 40μM concentration of NSC proliferated significantly less already at week 2 of the co-culture and at week 3 cells treated with 40μM NSC ceased to grow (Fig 1F). This indicated that primary BCR-ABL-expressing cells are highly dependent on RAC signaling, which is in direct agreement with our previous findings [40]. Subsequently, we wondered whether also primary BC CML cells are dependent more on RAC2 than RAC1 expression. To address this, we transduced CD34^+^ BC CML cells with lentiviral shRNA constructs and achieved efficient and specific downregulation of either RAC1 or RAC2 (S1 Fig). Transduced cells were sorted, plated on stroma and followed for 5 weeks of co-culture. Similarly to the BCR-ABL-transduced CB cells, primary BC CML cells also showed marked proliferative disadvantage upon downregulation of RAC2, with RAC1 depletion having little effect (Fig 1G). Overall, these data suggest that BCR-ABL-transformed cells critically depend on RAC2 for their proliferation and self-renewal.

### RAC2 depletion profoundly impairs expansion of primitive leukemic cobblestone cells

**Fig 2:**
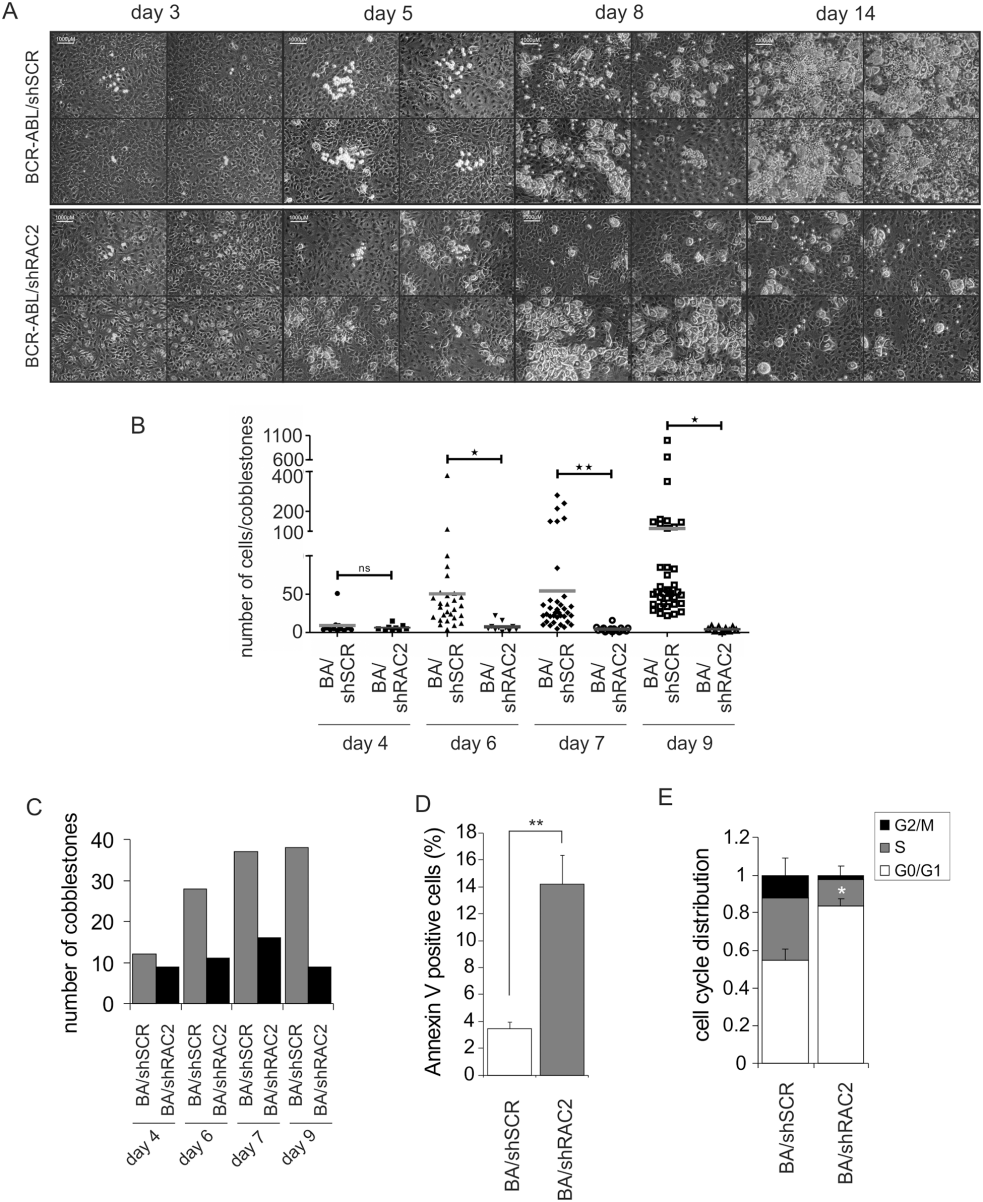
Depletion of RAC2 impairs expansion of primitive leukemic cobblestone cells. CB CD34^+^ stem/progenitor cells were double-transduced with BCR-ABL and either control scrambled shRNA vector (shSCR) or with RAC2-targeting shRNA vector (shRAC2) and 2X10^3^ double-transduced cells were plated on MS5 stromal cells, co-cultures were evaluated daily and images of cobblestones we acquired for 14 days (four representative images of each are shown in A). (B) The total number of cobblestones present in the culture of control or shRAC2-transduced BCR-ABL cells is shown for the indicated time points. (C) Acquired images of cobblestones were overlaid with a grid and the number of cells per individual cobblestone was counted for control and shRAC2-transduced cells, for the time points as in panel B. (D) CB CD34^+^ stem/progenitor cells were double-transduced with BCR-ABL and either control scrambled shRNA vector (shSCR) or with RAC2-targeting shRNA vectors (shRAC2) and 5x10^3^ double-transduced cells were sorted and plated on MS5 stromal cells. After 10 days of co-culture cobblestone cells were harvested and Annexin V staining was performed to measure apoptosis. The average of three independent experiments is shown. Error bars represent standard deviation. (E) Cobblestone cells from cultures like described in panel D were harvested and used for staining in hypotonic PI solution to evaluate cell cycle distribution. The average of three independent experiments is shown. Error bars represent standard deviation. * *P*<0.05, ** *P*<0.01.

To gain more insight into the effect of RAC2 depletion on the most primitive, cobblestone-forming leukemic cells, we performed extensive microscopical evaluation of BCR-ABL-transformed co-cultures on stroma. Interestingly, early during the co-culture both the number and appearance of RAC2-depleted cobblestones were not different than of control counterparts (Fig 2A and 2B). However, apparent from day 6 to 9 shSCR-transduced cobblestones increased in number and in size while shRAC2-transduced cells did not expand (Fig 2A–2C). From day 9 RAC2-deficient cobblestones started to be depleted from the co-culture and disappeared completely by day 14, suggesting impaired survival of these cells (Fig 2A). Disappearance of cobblestones during the co-culture and the dramatic decrease in cell counts (Fig 1A and 1B) suggested that the primitive BCR-ABL cells underwent cell death; therefore Annexin V FACS analyses were performed on the cobblestone fraction of BCR-ABL-transduced co-cultures. While the control-transduced cells displayed a low percentage of Annexin V-positivity, this percentage was significantly increased in RAC2-depleted cells (Fig 2D) suggesting that an increased apoptotic response was part of the RAC2-deficient phenotype. Next, we assessed the effect of RAC downregulation on the cell cycle status of the leukemic cobblestone cells. Upon RAC2 depletion, the fraction of cells in the S and G2/M phase of the cell cycle was markedly reduced (Fig 2E), which was in accordance with our microscopical observations (Fig 2A–2C).

### Depletion of RAC2 causes mitochondrial dysfunction in BCR-ABL-expressing HSPCs

**Fig 3:**
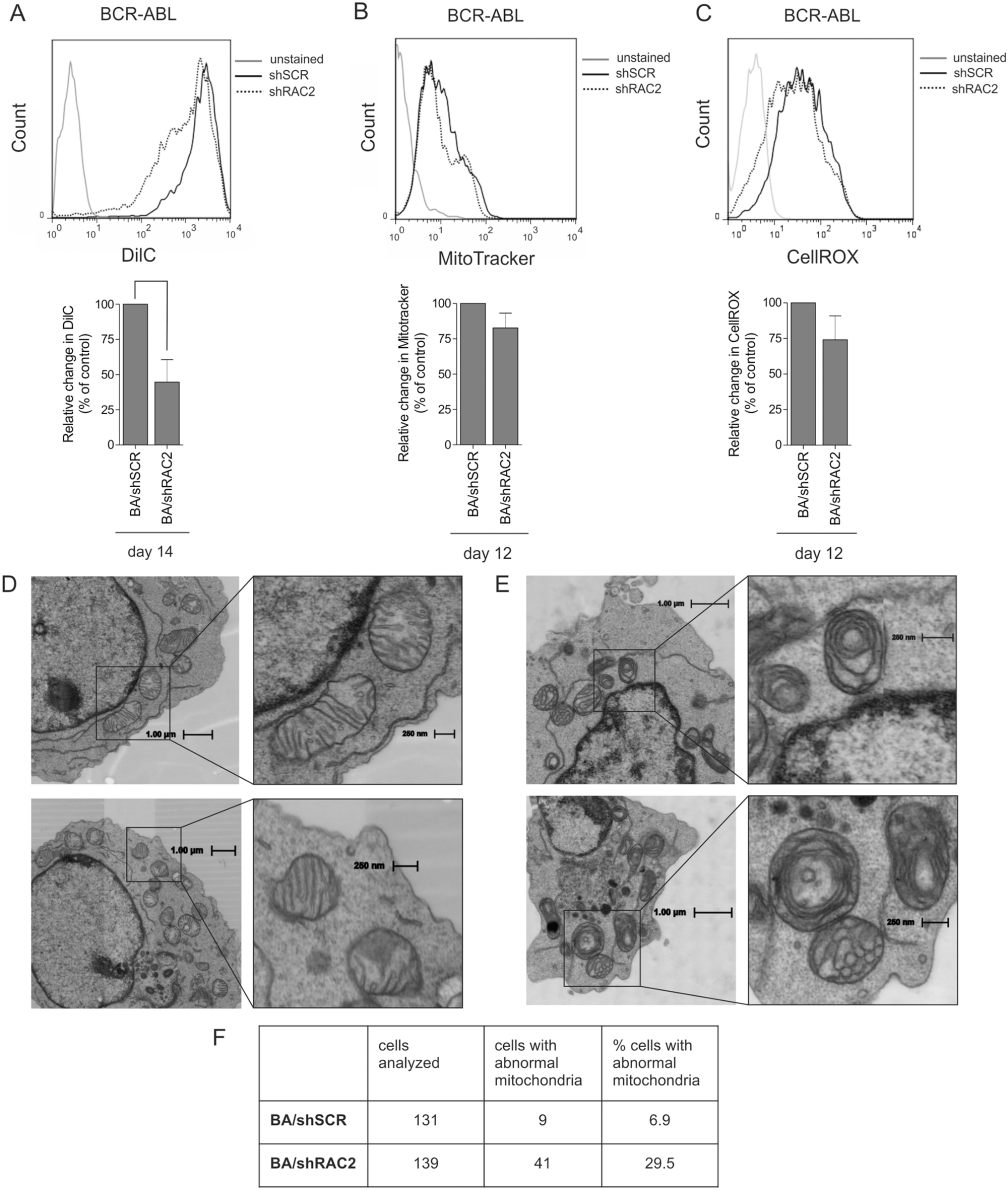
Depletion of RAC2 causes mitochondrial dysfunction in BCR-ABL-expressing HSPCs. (A) 5x10^3^ BCR-ABL and shSCR or shRAC2 double-transduced cells were sorted and plated on MS5 stromal cells. Suspension cells were harvested after 14 days of co-culture and stained with DilC to measure changes in mitochondrial membrane potential. Representative FACS plots are shown. Quantification of FACS measurements represented as changes in MFI relative to control is shown below. The average of four independent experiments is shown with standard deviation. (B) Suspension cells from the double-transduced co-cultures like described in panel A were harvested after 12 days and stained with MitotrackerRed to measure mitochondrial mass. A representative FACS plot is shown. Quantification of FACS measurements represented as MFI relative to control is shown below. Average of three independent experiments is shown with standard deviation. (C) Suspension cells from the double-transduced co-cultures (BCR-ABL and shSCR or shRAC2) were harvested after 12 days and stained with CellROX to measure oxidative stress. A representative FACS plot is shown. Quantification of FACS measurements represented as MFI relative to control is shown below. Average of three independent experiments is shown with standard deviation. (D) BCR-ABL and shRNA double-transduced cells expanded in stromal co-cultures for 7 days were harvested, pelleted and embedded in EPON, and ultrathin sections were analyzed by STEM. Large area scans of high resolution were acquired allowing evaluation of the ultrastructure of mitochondria in large number of control cells (D) or shRAC2-transduced cells (E), representative images of the abnormalities in the structure of mitochondria are shown). (F) 131 control and 139 shRAC2-transduced cells were analyzed for the presence of mitochondrial abnormalities (as seen in E) and the cells with aberrant mitochondria were enumerated. ** *P*<0.01.

We next questioned whether mitochondrial dysfunction might underlie the pheneotypes we observed in our human leukemia models upon RAC2 depletion. The mitochondrial membrane potential was assessed in human CD34^+^ CB cells transduced with BCR-ABL and shRAC2 vectors using DilC FACS analysis. Depletion of RAC2 caused a marked decrease in the mitochondrial membrane potential in BCR-ABL-transduced HSPCs (Fig 3A). The MitoTracker Red signal intensity was comparable between the control and RAC2-depleted BCR-ABL cells, suggesting that the mitochondrial mass was not changed (Fig 3B). Moreover, the level of ROS measured in BCR-ABL HSPCs was also reduced upon RAC2 downregulation, albeit the level of reduction did not reach statistical significance (Fig 3C). Overall, these results showed that RAC2 depletion negatively affected mitochondrial membrane potential in BCR-ABL-expressing cells, prompting us to investigate whether the ultrastructure of mitochondria was also altered. Therefore, we analyzed shRAC2-transduced BCR-ABL cells by electron microscopy (EM). We applied automated EM acquisition of large fields of view at high resolution, recently introduced as “Nanotomy” [38], which allowed an unbiased assessment of a large number of cells as wells as gaining insight into the ultrastructure of organelles. Analysis of acquired EM images revealed that RAC2-depleted cells displayed a particular shape of mitochondrial cristae, which were forming concentric structures very different from the usual, lamellar appearance of cristae (Fig 3D and 3E). These concentric structures were observed in almost one-third of RAC2-depleted cells, while only a few of the control cells contained such abnormal mitochondria (Fig 3F). Moreover, an altered mitochondrial structure could be seen in erythroid, myeloid and megakaryocytic precursors within the RAC2-deficient sample, suggesting that the mitochondrial aberrations occurred already in HSPCs and propagated across all the differentiation lineages (data not shown).

### RAC1 and RAC2 interact with distinct sets of proteins

**Fig 4:**
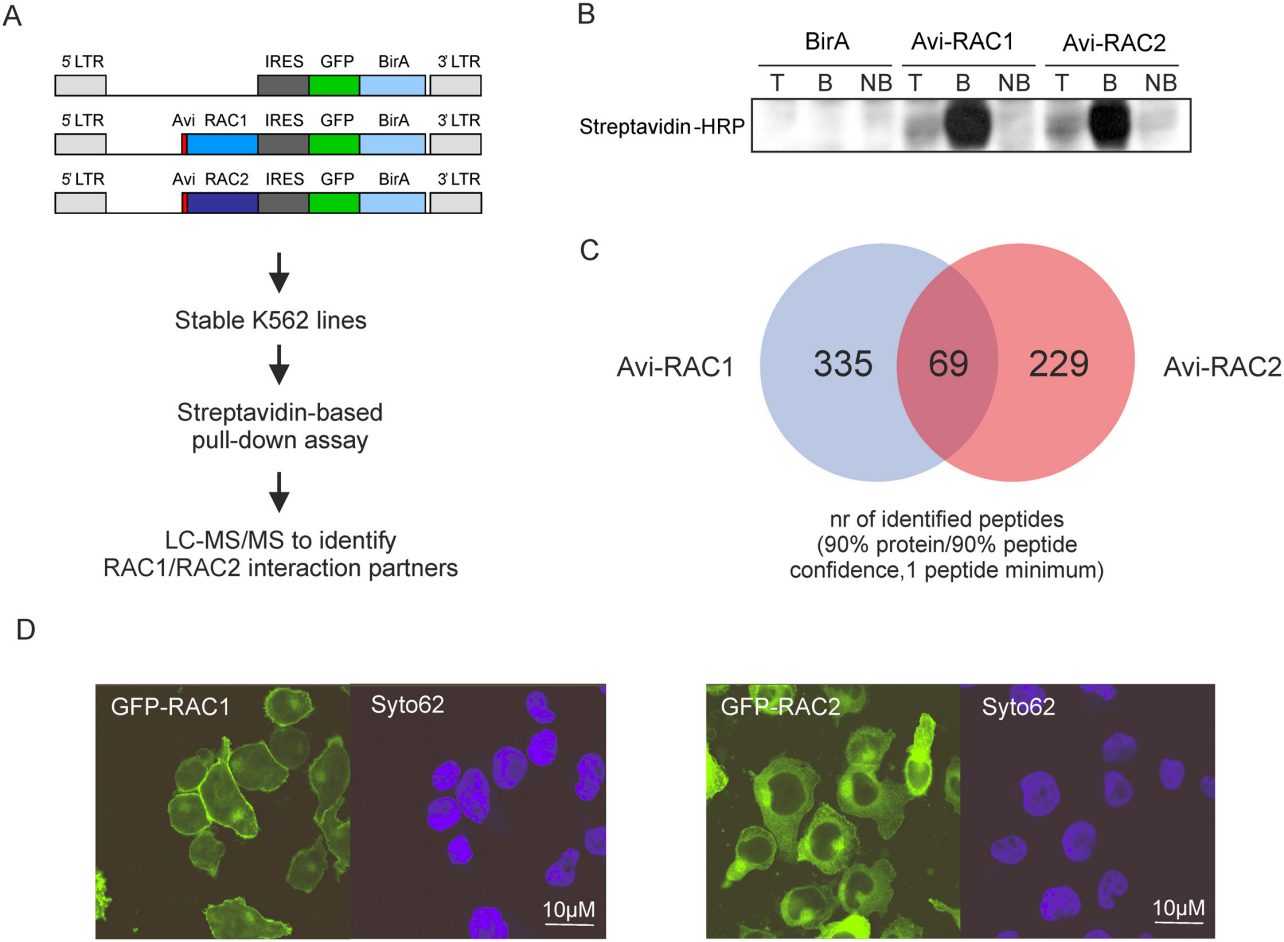
RAC1 and RAC2 interact with distinct sets of proteins. K562 cells were transduced with retroviral constructs containing Avi-tagged RAC1 (Avi-RAC1) or RAC2 (Avi-RAC2) and BirA biotin ligase, or BirA-only control construct (BirA) sorted, and streptavidin-based pull-down assay was performed. Bound fractions were then used for mass spectrometry analysis to identify RAC1- or RAC2-specific interaction partners (experimental setup shown in A). (B) Efficiency of the pull-down assay was assessed by Western blot detecting Avi-tagged RAC1 and RAC2. T: total cell lysate fraction; B: bound fraction; NB: non-bound fraction. (C) Mass spectrometry analysis identified 335 RAC1-specific and 229-RAC2-specific peptides using 90% protein/90% peptide confidence cut-off. (D) Cytospins of GFP-RAC1- or GFP-RAC2-transduced TF-1 cells were analyzed for RAC localization. Syto62 was used to stain the nucleus.

The results of shRNA interference experiments suggested that RAC2 would have specific functions in human leukemic cells crucial for their survival and long-term proliferation, possibly linked to mitochondria. To further explore these functions in an unbiased way, we performed an open-minded proteomics screen to identify specific interaction partners of the two RAC proteins, using methodology that we successfully used previously to study the complex composition of the Polycomb Repressive Complex 1 [39]. To this end, we transduced K562 cell line with retroviral constructs containing Avi-tagged RAC1 or RAC2 as well as biotin ligase that allowed *in vivo* biotinylation and efficient pull-down of RAC1- or RAC2-associated protein complexes (Fig 4A and 4B), The pull-down fractions were then analyzed by tandem mass spectrometry to identify RAC1 or RAC2 associated proteins (experimental setup shown in Fig 4A). Consequently, 335 RAC1-specific and 229 RAC2-specific peptides were identified (Fig 4C). GO annotation revealed that while the common interactome of RAC1 and RAC2 comprised mainly of cytoskeleton proteins, their specific interaction partners fell into distinct categories. RAC1 associated with a significant set of plasma membrane proteins, while RAC2 interacted with a group of mitochondrial membrane proteins forming mitochondrial transport complexes, as well as mitotic spindle proteins (Table 1). Interaction with such different sets of proteins suggested differential subcellular localization of RAC1 and RAC2. Indeed, when we transduced TF-1 leukemic cell line with GFP-tagged constructs of either RAC1 or RAC2, we could observe that while RAC1 was strongly enriched in the plasma membrane, RAC2 was localized in the cytoplasm and enriched in the perinuclear region (Fig 4D and S1 Movie and S2 Movie).

**Table 1:** MS-identified specific interaction partners of RAC1 and RAC2*.

Term	Count	PValue	Genes
RAC1 interactome			
GO:0005856 cytoskeleton	41	8.47E-06	DNAH11, LZTS1, KRT6B, CALD1, SLC38A6, TTLL4, AKAP9, CD2AP, CDSN, LATS2, DNAH5, VCL, RPA1, CEP250, AVIL, KRT85, EMD, FGD4, NES, KIF12, EPPK1, TANC1, CENPF, SYNPO2, RACGAP1, EML6, GRM1, JUP, ARPC1B, CCDC6, SYNE2, EPB41L1, CASS4, KIF1B, LASP1, TPPP, SPTBN2,
GO:0044430 cytoskeletal part	27	9.83E-04	DNAH11, KRT6B, LZTS1, CALD1, TTLL4, AKAP9, CD2AP, DNAH5, LATS2, CEP250, KRT85, EMD, KIF12, NES, TANC1, CENPF, RACGAP1, GRM1, EML6, ARPC1B, KIF1B, LASP1, TPPP, SPTBN2, PLA2G6, SPTB, SPTAN1
GO:0015629 actin cytoskeleton	11	5.20E-03	JUP, ARPC1B, LASP1, CALD1, AVIL, SPTBN2, SYNPO2, CD2AP, SPTAN1, SPTB, VCL
GO:0070161 anchoring junction	8	1.16E-02	JUP, CASS4, LASP1, DSP, CDSN, MLLT4, SPTAN1, VCL
GO:0005912 adherens junction	7	2.35E-02	JUP, CASS4, LASP1, DSP, MLLT4, SPTAN1, VCL
GO:0015630 microtubule cytoskeleton	14	5.01E-02	DNAH11, KIF12, TTLL4, CENPF, AKAP9, RACGAP1, EML6, LATS2, DNAH5, KIF1B, CEP250, TPPP, PLA2G6, EMD
RAC2 interactome			
GO:0005741 mitochondrial outer membrane	6	1.34E-03	DNM1L, SAMM50, MTX1, NLRX1, GK, PPP1CC
GO:0015630 microtubule cytoskeleton	14	1.40E-03	KIF23, PCNT, CEP78, DNAH3, DNAH2, AURKB, KIAA1009, MAD2L1BP, NAV1, MAP4, TUBG1, MAP6, DST, MYH10
GO:0005856 cytoskeleton	23	6.01E-03	KIF23, PCNT, SSH3, CEP78, MYH2, DNAH3, MYLK2, AURKB, DNAH2, CAPZB, KIAA1009, CORO1C, SYNE1, MAD2L1BP, NAV1, GOPC, MAP4, TUBG1, MAP6, SEPT6, DST, MYH7B, MYH10
GO:0005819 spindle	6	1.09E-02	KIF23, MAD2L1BP, AURKB, TUBG1, MYH10, KIAA1009
GO:0005739 mitochondrion	18	1.86E-02	ALDH18A1, NOL3, DNM1L, SAMM50, MTX1, MRPS5, IDH3B, NLRX1, PPP1CC, TIMM23, PCK2, MTRR, GLYAT, AGPAT5, PARS2, GK, AP2M1, LRP5
GO:0031966 mochondrial membrane	8	6.73E-02	ALDH18A1, DNM1L, SAMM50, MTX1, NLRX1, GK, PPP1CC, TIMM2

*K562 cells were transduced with retroviral constructs containing Avi-tagged RAC1 (Avi-RAC1) or RAC2 (Avi-RAC2) and BirA biotin ligase, or BirA-only control construct (BirA) sorted, and streptavidin-based pull-down assay was performed. Bound fractions were then used for mass spectrometry analysis to identify RAC1- or RAC2-specific interaction partners. GO annotation was performed on the MS identified proteins and the most significant GO terms for RAC1 and RAC2 are presented in the table.

### The RAC2-interacting protein SAM50 is required for long-term expansion of human BCR-ABL-expressing HSPCs

**Fig 5:**
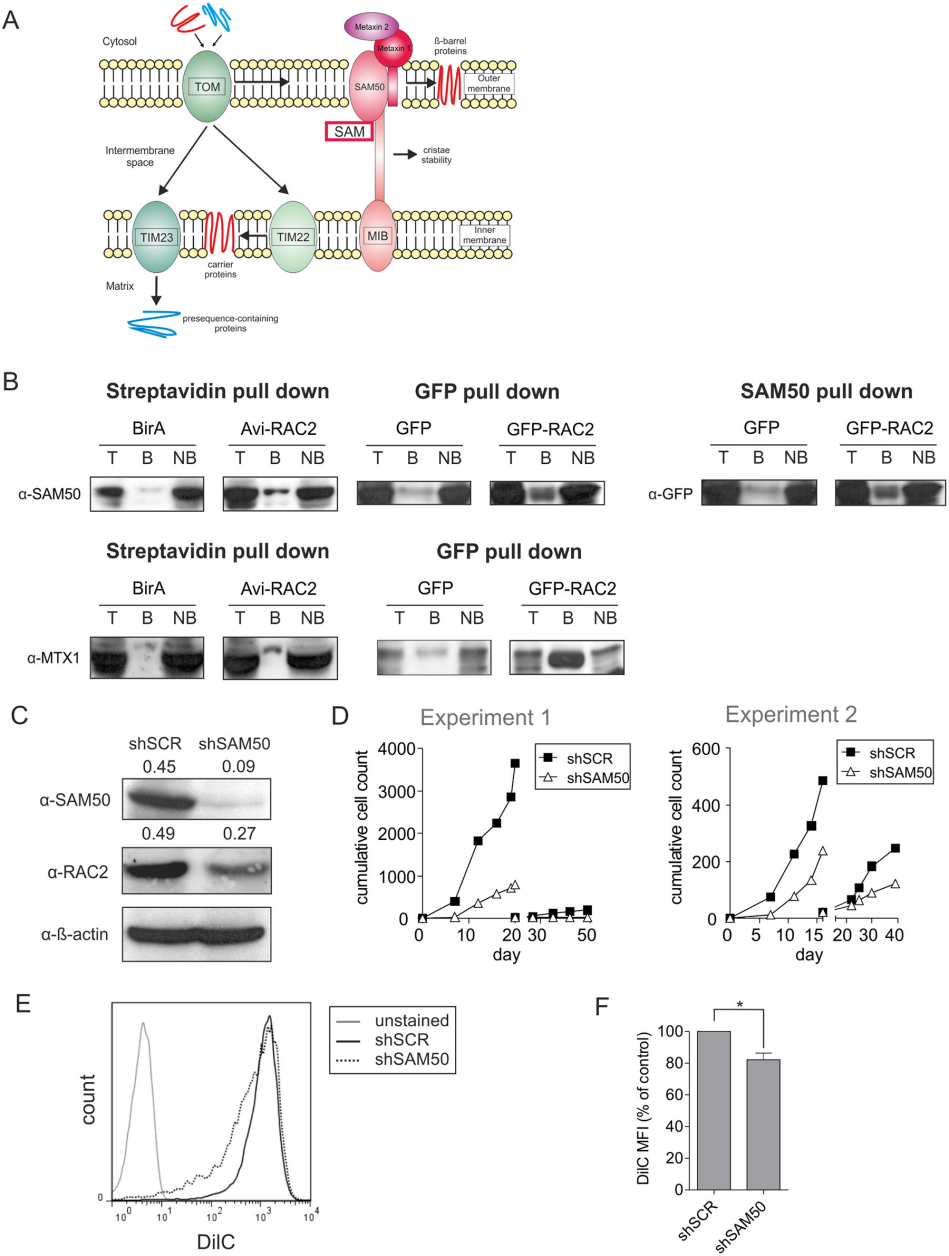
RAC2 interaction with mitochondrial transport proteins is required for the long-term expansion of human BCR-ABL-expressing HSPCs. (A) Schematic representation of the mitochondrial transport complexes and their function. (B) K562 cells transduced with retroviral constructs containing Avi-tagged RAC2 (Avi-RAC2) and BirA biotin ligase, or BirA-only control construct (BirA) were sorted, and streptavidin-based pull-down assay was performed. Conversely, immunoprecipitation with anti-SAM50 antibody was performed. Alternatively, K562 cells were transduced with lentiviral GFP-tagged RAC2 (GFP-RAC2) and pull-down assay using GFP-affinity beads was performed. Empty GFP vector-transduced cells were used as a control. Efficiency of pull-down assay was assessed by Western blot detecting either SAM50 or Metaxin 1 (Streptavidin and GFP pull-down), or GFP (SAM50 pull-down). T: total cell lysate fraction; B: bound fraction; NB: non-bound fraction. (C) K562 cells transduced with the control scrambled shRNA vector (shSCR) or with the SAM50-targeting shRNA vector (shSAM50) were sorted and used for Western blot analysis to determine SAM50 protein levels. The quantification of protein expression normalized to control is indicated above each lane. (D) CB CD34^+^ stem/progenitor cells were double-transduced with BCR-ABL and either control shSCR or with shSAM50. 5*10^3^ double-transduced cells were sorted per group and plated on MS5 stromal cells. Cultures were demi-depopulated on indicated days for analysis and replated where indicated. Cumulative cell growth is shown for two representative experiments of 4 independent experiments. (E) 5*10^3^ BCR-ABL and shSCR or shSAM50 double-transduced cells were sorted and plated on MS5 stromal cells. Suspension cells were harvested after 14 days of co-culture and stained with DilC to measure changes in mitochondrial membrane potential. Representative FACS plots are shown. (F) Quantification of FACS measurements as described in panel (E) represented as changes in MFI relative to control is shown below. Average of four independent experiments is shown with standard deviation. * *P*<0.05.

The proteomics screen identified several mitochondrial transport proteins as interaction partners of RAC2. Two of those proteins, SAM50 and Metaxin 1 function within the SAM complex, involved in incorporation of proteins to the mitochondrial outer membrane, as well as regulating cristae stability (Fig 5A) [41,42]. We verified the interaction between these two mitochondrial proteins and RAC2 using Streptavidin-based and GFP-based pull-down strategies. In both cases, SAM50 and Metaxin 1 were enriched in the RAC2 pull-down fraction above the control background levels. Moreover, imunoprecipitation with anti-SAM50 antibody confirmed enrichment of RAC2 signal in the SAM50 pull-down fraction (Fig 5B). Subsequently, we functionally tested the role of SAM50 in human leukemic HSPCs. To this end, we generated a lentiviral shRNA construct that efficiently downreguated SAM50 (Fig 5C, top panel). Interestingly, this decrease in the protein level of SAM50 was accompanied by a decrease in the levels of RAC2, although less pronounced (Fig 5C, middle panel). Next, we simultaneously transduced CD34^+^ CB cells with the retroviral BCR-ABL construct and the lentiviral shRNA-containing constructs, double-transduced cells were then sorted and plated on stroma for long-term co-cultures. Similarly to RAC2-depleted cells, SAM50-deficient BCR-ABL HSPCs also showed a marked proliferative disadvantage in stromal co-cultures and a diminished replating capacity (Fig 5D). Moreover, the mitochondrial membrane potential of shSAM50-transduced cells was significantly reduced (Fig 5E and 5F). Taken together, this data suggest that disturbed interactions between RAC2 and mitochondrial transport proteins cause mitochondrial dysfunction resulting in impaired proliferation and enhanced apoptosis phenotypes observed in RAC2-depleted human BCR-ABL cells.

## Discussion

Although over the past years it has become clear that RAC proteins fulfill important roles in the hematopoietic compartment, our insight into molecular mechanisms downstream of RAC1 and RAC2 is still limited. Here, we show that BCR-ABL-transformed human hematopoietic stem/progenitor cells (HSPCs) critically depend on RAC2 for their proliferation and survival, which is linked to maintaining an appropriate mitochondrial integrity and mitochondrial membrane potential. RAC2 specifically interacted with a set of mitochondrial proteins including SAM50 and Metaxin1, and downregulation of SAM50 also impaired the proliferation and replating capacity of BCR-ABL-expressing cells, again associated with a decreased mitochondrial membrane potential.

Our data are in line with studies on the role of RAC GTPases in murine leukemia models that identified the importance of those proteins for the establishing and maintenance of the disease [29–33,43]. In particular, RAC2 was shown to be critical for the development of BCR-ABL-mediated malignancy [30,44]. This strong dependency was also evident in our long-term cultures of BCR-ABL-transduced human HSPCs, where RAC2-, but not RAC1-depleted cells showed strongly reduced proliferation, reduced progenitor and stem cell frequencies and a diminished replating capacity. In line with our findings in oncogene-transduced human CB models, we observed that primary human CB CML cells were also specifically dependent on RAC2 for their long-term proliferation. Although studies on a wider collection of leukemic samples are necessary, these results are a first validation that RAC2 is necessary for primary human BCR-ABL-induced leukemia as well.

Stromal co-cultures model several aspects of the interactions between HSCs and the bone marrow environment, such as homing, adhesion, retention and long-term proliferation. Therefore, we utilized this system to gain insight into the dynamics of the phenotype elicited by RAC2 depletion in human BCR-ABL HSPCs. The initial migration and adhesion to stroma were not affected, and early RAC2-deficient cobblestones were indistinguishable from their control counterparts. However, from day 6 onwards the changes in cobblestone size became apparent, whereby RAC2-depleted cells stopped proliferating and ultimately underwent apoptosis. This is in agreement with the previous reports from murine models, in which loss of Rac2 prevented the development of BCR-ABL-initiated leukemia by increasing apoptosis of LSCs, rather that altering their interactions with the microenvironment [32]. Recently, Nieborowska-Skorska *et al*. showed that RAC2 altered mitochondrial membrane potential and electron flow through mitochondrial respiratory chain (MRC) complex III. Consequently, high levels of reactive oxygen species were generated that were responsible for genomic instability of CML cells and progression to the blast crisis. This effect was counteracted by either knockdown of RAC2 or pharmacological targeting of RAC activity with small molecule inhibitor [44]. In our experiments we observed a significant decrease in mitochondrial membrane potential in RAC2-deficient BCR-ABL cells, which together with the unchanged mitochondrial mass was indicative for defective functioning of mitochondria. Moreover, upon RAC2 downregulation, ROS levels within the BCR-ABL-transduced cells decreased, which is in agreement with previous findings [44]. These functional changes were paralleled by an altered ultrastructure of mitochondria in RAC2-deficient BCR-ABL cells. In shRAC2-transduced cells, mitochondrial cristae displayed a particular, circular shape, which was almost completely absent from the control cells. This aberrant shape of cristae was previously found to be associated with mitochondrial dysfunction [41,42,45,46]

Murine knock-out models have showed that despite the very high amino acid sequence homology, RAC1 and RAC2 have non-redundant functions in hematopoietic cells, which could be explained at least in part by the differences in the C-termini of the two proteins leading to their distinct subcellular distribution in murine neutrophils [47]. The main function of RAC GTPases is traditionally associated with cytoskeleton rearrangements and thus processes such as regulation of cell shape, migration and adhesion [27,28,48]. Accordingly, the common part of the interactome identified in our proteomics screen comprised of cytoskeleton proteins. In agreement with the microscopical observation that RAC1 localized in the plasma membrane, RAC1-specific interaction partners were GO annotated as anchoring junction proteins. Conversely, within RAC2-associated complexes several mitochondrial proteins were identified. This, together with the functional and structural defects of mitochondria in RAC2-depleted cells prompted us to study in more detail the mitochondrial interactome of RAC2. Three of the identified RAC2 interactions partners were annotated as mitochondrial membrane proteins that constitute the mitochondrial transport complexes and two of them, SAM50 and Metaxin 1, are known to interact with each other within the SAM complex [41,42]. Here, we confirmed that SAM50 and Metaxin 1 co-precipitate with either Avi-tagged or GFP-tagged RAC2. Although some background levels were detected in control precipitates, both SAM50 and Metaxin 1 were clearly enriched in the RAC2-bound fraction. Moreover, for SAM50 the interaction was confirmed by SAM50-specific immunoprecipitation. Also at the functional level, downregulation of SAM50 in BCR-ABL-expressing HSPCs caused a marked proliferative disadvantage and decreased replating capacity, in line with the RAC2-knockdown phenotype. Interestingly, knockdown of SAM50 also resulted in slightly reduced levels of RAC2 (Fig 5C). We speculate that the disruption of RAC2-SAM50 complex by downregulation of SAM50 could also affect RAC2 stability to some extent, leading to an increase in protein degradation and consequently slightly lower RAC2 protein levels, although further studies are required to resolve this. A decrease in mitochondrial membrane potential was also detected, albeit to a lesser extent than seen upon RAC2 downregulation. Possibly, this is related to the observation that RAC2 interacts with more than one mitochondrial membrane protein which would explain why depletion of RAC2 had a more profound effect on mitochondrial membrane potential than downregulation of SAM50 alone.

Recent studies showed that depletion of SAM50 and another mitochondrial membrane protein, mitofilin, resulted in disorganized cristae, morphologically similar to the phenotype we observed upon RAC2 downregulation [41,45,46]. Interestingly, depletion of these proteins resulted not only in a deficient assembly of the MRC complexes, but it also altered the expression of several genes involved in oxidative phosphorylation [41]. Deregulated expression of MRC components has recently been shown to be characteristic for the LSCs in CML chronic phase, when compared with normal HSCs [49]. The altered expression pattern indicated an increased level of oxidative phosphorylation in CML LSCs cells that could reflect their increased proliferation rate [49].

In conclusion, we propose that the active metabolic state of BCR-ABL-expressing cells renders them highly dependent on the proper functioning of mitochondria and that mitochondrial dysfunction caused by RAC2 downregulation results in depletion of these cells from the *in vitro* cultures. Although more experiments are needed to determine the exact molecular mechanisms, the interaction between RAC2 and mitochondrial transport proteins identified here provides a intriguing novel link between RAC2 and mitochondrial morphology and function.

## Supporting Information

S1 Fig (A) Cord blood (CB) CD34^+^ stem/progenitor cells were transduced with control scrambled shRNA vector (shSCR) or with RAC1/RAC2-targeting shRNA vectors (shRAC1 or shRAC2), sorted, cultured for 10 days on stroma and used for RNA extraction. Quantitative PCR was performed to measure RAC1 (left panel) or RAC2 mRNA levels (right panel) normalized against RPL27 mRNA. Alternatively, cells were used for Western blot analysis to determine RAC1 or RAC2 protein levels. The quantification of protein expression normalized to control is indicated above each lane. (B) BC CML CD34^+^ stem/progenitor cells were transduced with control scrambled shRNA vector (shSCR) or with RAC1/RAC2-targeting shRNA vectors (shRAC1 or shRAC2), sorted, cultured for 10 days on stroma and used for RNA extraction. Quantitative PCR was performed to measure RAC1 (top panel) or RAC2 mRNA levels (bottom panel) normalized against RPL27 mRNA.
(TIF)

S1 Movie (AVI)

S2 Movie (AVI)
